# Trial of a Physician

**Published:** 1848

**Authors:** 


### ARTICLE XIV.

### *Trial of a Physician for Assault and Battery.*

A trial of a novel character has lately taken place in N. H., in which a very respectable physician was the defendant. Trials of medical men for *mal-practice* have not been very uncommon, and have afforded opportunities for unprincipled individuals to attempt to obtain money from those who had used their time and best skill for the relief of the afflicted. The case alluded to is a new mode of harassing the profession; and, it would seem, with exactly not the same object in view---as criminal punishment, instead of pecuniary damages, was the penalty anticipated. The complaint was made by L. C. Delaware and his wife, of South Hampton, N. H., against Dr. J. B. Gale, of Salisbury Mills, Mass.---the charge against the latter being for an assault and battery upon Mrs. D. in March, 1847, upon the occasion of her confinement with her first child. It would seem that this worthy couple, since the alleged assault more than a year ago, have, through the means of certain books which have been freely circulated respecting the impropriety of employing male accoucheurs in midwifery cases, or by some other meaus, become fully convinced of the gross indelicacy of snch a practice, and for the purpose of showing their abhorrence of it, and to prevent as far as possible its repetition, the present complaint was made. How far they were justified, by the circumstances of the case, in adopting such a course, the evidence given on the trial will show. The following is the testimony of Mrs. D. herself, while that of Mrs. Woodman, an intelligent woman who was present, did not differ much from it.

"Dr. J. B. Gale, on the morning of the 5th of March, 1847, called to see me, agreeably to my request, which was conveyed to him by my husband. I was sick at that time, and expected to be delivered of a child. Dr. Gale came into my room about 5 o'clock and staid till 9, when I was delivered of a child, my first born. My mother, and Mrs. Woodman were in the room when the doctor came, and remained there until the child was born. He had been in the room about fifteen minutes, when he came to where I was lying upon the bed, and after remarking---'Sister, you are doing well---don't be scared,' he commenced making an assault upon me by placing his hands upon my person. I had labor pains occasionally, and at intervals of a quarter and a half hour, he renewed his assaults, by placing his hand upon my person. At these different times, I told the doctor to let me alone, and to go away, but he did not. I also asked for my husband: but the doctor replied, "Umph! you do not need your husband." The doctor did not ask my consent to make an examination. I think he increased my pains at each examination he made. I lay upon my left side---the doctor came up and made his examinations until my child was born, which was at 9 o'clock.

"On the cross-examination, the evidence of Mrs. D. did not vary materially from that of the examination in chief—and was as follows:---While lying upon the bed, my person was not exposed, to my knowledge---the doctor remained at my side five or ten minutes at a time---he assisted at the birth of the child. I did not think it was right *that the doctor should handle me*---and told my husband so. This affair happened sixteen months since, and the reason I had not complained of the doctor before, was because I *wanted time to think of it!* The doctor visited me twice afterwards, and promised to call again, but did not."

Several physicians were called for the defence, who testified that the conduct of Dr. G., as stated by these witnesses, was not different from the usual practice on such occasions. After the address of the counsel for the government, Justice Currier, before whom the case was tried, briefly reviewed the evidence, and after remarking that there appeared to be no cause for the complaint, that Dr. Gale, in doing his duty, had committed no offence whatever, ordered him to be discharged.

